# Decontamination of T-2 Toxin in Maize by Modified Montmorillonite Clay

**DOI:** 10.3390/toxins11110616

**Published:** 2019-10-24

**Authors:** Bunmi K. Olopade, Solomon U. Oranusi, Obinna C. Nwinyi, Isiaka A. Lawal, Sefater Gbashi, Patrick B. Njobeh

**Affiliations:** 1 Department of Biological Sciences, Covenant University, Ota 112233, Ogun State, Nigeria; solomon.oranusi@covenantuniversity.edu.ng (S.U.O.); obinna.nwinyi@covenantuniversity.edu.ng (O.C.N.); 2 Department of Biotechnology and Food Technology, University of Johannesburg, Doornfontein Campus, Gauteng 2028, South Africa; sefatergbashi@gmail.com (S.G.); pnjobeh@uj.ac.za (P.B.N.); 3 Chemistry Department, Faculty of Applied and Computer Science, Vaal University of Technology, Vanderbijlpark Campus, Boulevard, Vanderbijlpark 1900, South Africa; lawalishaq000123@yahoo.com

**Keywords:** decontamination, adsorption, T-2 toxin, montmorillonite clay, *Cymbopogon citratus*, maize

## Abstract

Montmorillonite clay has a wide range of applications, one of which includes the binding of mycotoxins in foods and feeds through adsorption. T-2 toxin, produced by some *Fusarium, Myrothecium,* and *Stachybotrys* species, causes dystrophy in the brain, heart, and kidney. Various formulations that include lemongrass essential oil-modified montmorillonite clay (LGEO-MMT), lemongrass powder (LGP), montmorillonite clay washed with 1 mM NaCl (Na-MMT), montmorillonite clay (MMT), and lemongrass powder mixed with montmorillonite clay (LGP-MMT) were applied to maize at concentrations of 8% and 12% and stored for a period of one month at 30 °C. Unmodified montmorillonite clay and LGP served as the negative controls alongside untreated maize. Fourier Transform Infrared (FTIR) spectra of the various treatments showed the major functional groups as Si-O and -OH. All treatment formulations were effective in the decontamination of T-2 toxin in maize. Accordingly, it was revealed that the inclusion of Na-MMT in maize at a concentration of 8% was most effective in decontaminating T-2 toxin by 66% in maize followed by LGP-MMT at 12% inclusion level recording a 56% decontamination of T-2 toxin in maize (*p* = 0.05). Montmorillonite clay can be effectively modified with plant extracts for the decontamination of T-2 toxin.

## 1. Introduction

**Figure 1:**
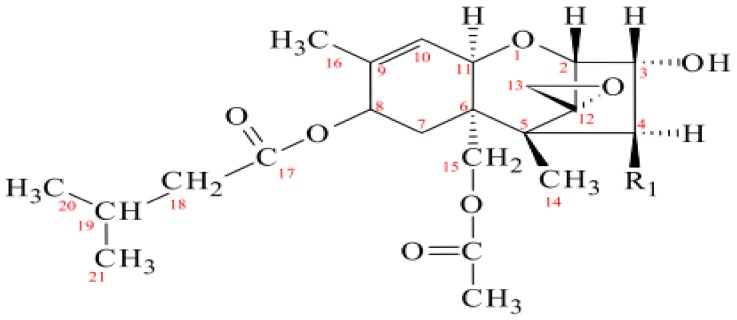
Chemical structure of T-2 toxin (R1 = OAc) adapted from European Food Safety Authority (2011).

Maize is grown worldwide, with the United States, China, and Brazil ranked as the leading producing countries contributing approximately 563 of the 717 million metric tonnes per year [1]. However, maize, like other cereals, is susceptible to contamination by mycotoxins, including T-2 toxin. T-2 toxin is common in grains, including oats, soybeans, rice, barley, maize, and wheat [2]. *Fusarium langsethiae* and *F. sporotrichioides* are some of the *Fusarium* species that infect crops in the field and storage during which time they produce T-2 toxin [3]. This toxin belongs to the family of mycotoxins known as the trichothecenes produced mainly by *Fusarium sporotrichioides, F. graminearum, F. poae,* and *F. culmorum* and causing diarrhea, inflammation, and emesis [4,5]. They can be grouped into Type A, B, C, and D, with T-2 toxin belonging to Type A trichothecenes, the most toxic group of trichothecenes. Trichothecenes are among the mycotoxins associated with human and veterinary diseases [6]. Like T-2, all trichothecenes have a common tetracyclic sesquiterpenoid 12,13-epoxytrichothec-9-ene ring system together with an epoxide group located at their C12, C13 positions, which accounts for their toxicity [7]. T-2 toxicity is induced via oxidative stress-mediated deoxyribonucleic acid (DNA) damage and apoptosis [8]. The chemical structure of T-2 toxin is shown in Figure 1.

T-2 toxin is a recognized inhibitor of protein synthesis, which occurs by the binding of peptidyl transferase, present in the 60S ribosomal subunit [9]. T-2 poisoning in man results in alimentary toxic aleukia (ATA). The toxin also causes ulcers as well as necrosis in the digestive tract, hemorrhagic inflammation, and dystrophy in the brain, heart, kidney, and liver [10]. The walls of blood vessels are damaged, provoking hemorrhagic diathesis [10]. T-2 also affects animals, with monogastric animals being more susceptible than ruminants. This is because ruminants, such as cattle, possess a rumen where de-acetylation and de-epoxidation of the toxin take place, thus limiting its toxicity [11]. The toxin also affects poultry, causing ulcerative and necrotic lesions as well as oral lesions, thereby leading to refusal of feed [12]. Studies have revealed that T-2 toxin is easily absorbed in the intestinal tract and is toxic to the organs of animals affecting the digestive system and liver of animals [13,14].

Decontamination processes involving physical, biological, and chemical methods are frequently required for the removal, destruction, and decontamination of mycotoxins [15]. Biological approaches include the activity of microorganisms (algae, bacteria, filamentous fungi, and yeasts) against mycotoxins using antibiosis or competition for nutrients and space [15]. Chemical approaches help to degrade mycotoxins by various gases, aldehydes, oxidizing agents, acids, and bases. The EC supports the application of physical methods and sorting procedures for decontaminating mycotoxins [16]. However, neither the mixing of batches to reduce mycotoxins to levels below the maximum tolerable limits nor decontamination using chemicals is permitted by the European Union [16]. Physical methods of detoxification include irradiation (gamma or ultraviolet irradiation), solvent extraction, inactivation by heat, and the use of mineral adsorbents (mainly clays) [17]. The disadvantages of food irradiation are the high costs involved and loss of vitamins (avitaminosis), while inactivation by heat may lead to a loss in nutritional properties of the commodities [18]. The use of mineral adsorbents, such as clay, is a cheaper and safer alternative to these methods, revealing their mycotoxin decontamination properties. Clays also serve as anti-caking agents in animal feed, and they are highly regarded as effective mycotoxin binding agents in animal feeds by enterosorption [19]. Enzymes such as oxidoreductases and hydrolases have also been thoroughly explored in the mitigation of mycotoxins in animal feed [20].

Montmorillonite clay has pharmaceutical applications where it serves as an excipient in drugs for oral or topical use [21]. The cytotoxicity of montmorillonite clay is low both in vitro and in vivo [22]. A study by Wei et al. [23] showed that modified hydrated sodium calcium aluminosilicate (HSCAS) adsorbent could be used in vivo to reduce toxicity induced by T-2 toxin. Therefore, clay can be safely expended in the storage of cereal crops for mycotoxin decontamination purposes. Montmorillonite, when compared with other nanoclays, has an advantage because it is abundant and environmentally friendly [24]. Each layer of montmorillonite has a lateral dimension of 200–600 nm. It is made up of an octahedral sheet sandwiched between two tetrahedral sheets. The arrangement of the sheets is such that the silicon oxide tetrahedron (SiO_4_) shares three out of four of its oxygen atoms with the central octahedral sheets [24]. Among various clay minerals such as kaolinite, illite, and chlorite, montmorillonite is more ideal for adsorption studies since it has a high cation exchange capacity, swelling capacity, and high surface area [25,26].

The use of plant extracts may have ancillary effects, thus preventing fungal growth and the production of mycotoxins. The oils of plants such as anise, basil, cinnamon, lemon, clove, lemongrass, spearmint, and oregano have been shown to inhibit fungi that produce DON, FB_1_, and ZEA [27]. *Cymbopogon citratus* (lemongrass) has been expended in the decontamination of *Aspergillus* species and limit AF production in maize [28]. Traditionally, lemongrass tea is consumed in South America, Asia, and West Africa, having anti-fever, anti-inflammatory, antiseptic, anti-dyspeptic, and carminative properties [29] with no toxicity reported both in animal and man. Fandohan et al. [30] revealed that the lethal dose (LD50) for lemongrass is >3500 mg/kg bw and is likely to cause hepatocyte necrosis, leukocytes infestation of liver parenchyma, and alteration of stomach structure in rats. Therefore, extracts and powder of *Cymbopogon citratus* can be safely used in the decontamination of mycotoxins in cereals. The aim of this study, therefore, was to decontaminate T-2 toxin in maize using formulations from montmorillonite clay together with extracts and powder of *Cymbopogon citratus*.

## 2. Results and Discussion

### 2.1. Scanning Electron Microscopy

**Figure 2:**
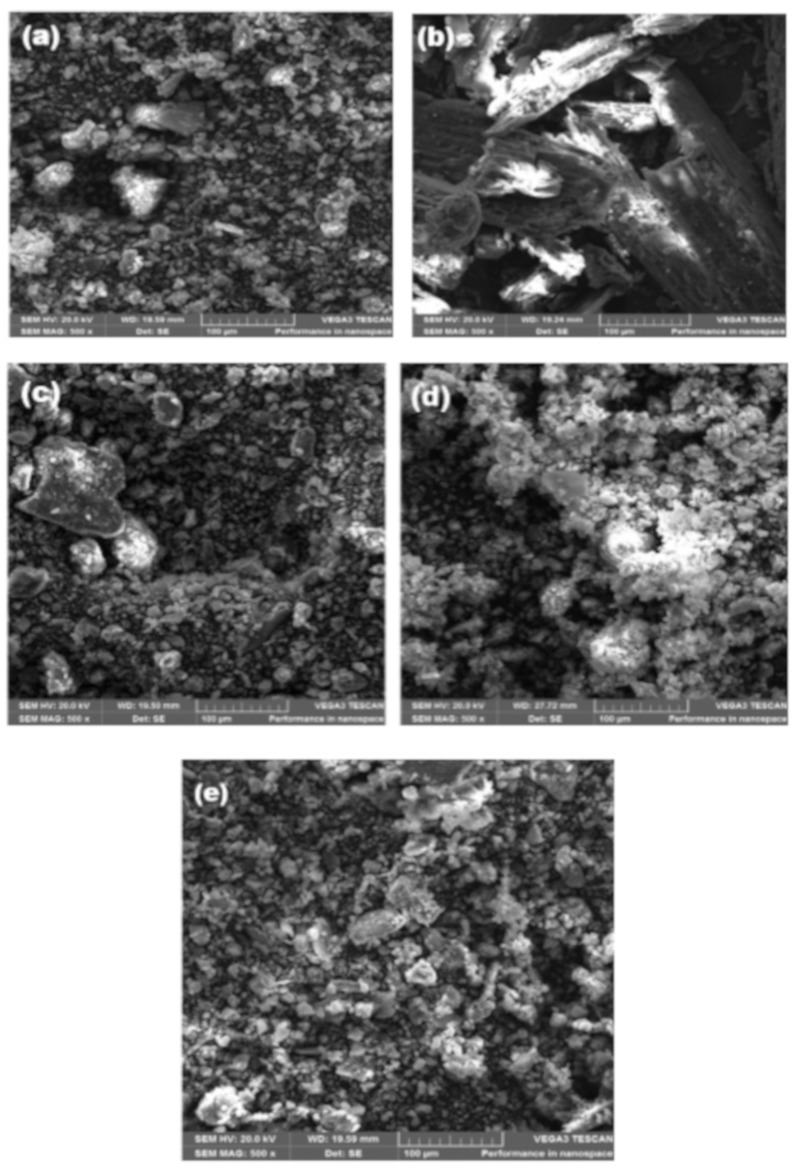
(**a**) Montmorillonite clay (MMT); this figure was published previously in Olopade et al. (2019). (**b**) Lemongrass powder (LGP); this figure was published previously in Olopade et al. (2019). (**c**) Montmorillonite clay washed with NaCl (Na-MMT); this figure was published previously in Olopade et al. (2019). (**d**) Montmorillonite clay with lemongrass essential oil (LGEO-MMT); this figure was published previously in Olopade et al. (2019). (**e**) Montmorillonite mixed with Lemongrass powder (LGP-MMT); this figure was published previously in Olopade et al. (2019).

The scanning electron micrographs of the various treatment formulations with a magnification of 500× and scale bar of 100 µm are shown in Figure 2a–e. The morphologies of the various formulations were similar and appeared as agglomerates. Unmodified montmorillonite (MMT) and Na-MMT had very similar morphology, while LGEO-MMT appeared more agglomerated than the other treatments because of fatty acids present in the crude extracts of *Cymbopogon citratus*.

### 2.2. Fourier Transform Infrared (FTIR) Spectroscopy

**Figure 3:**
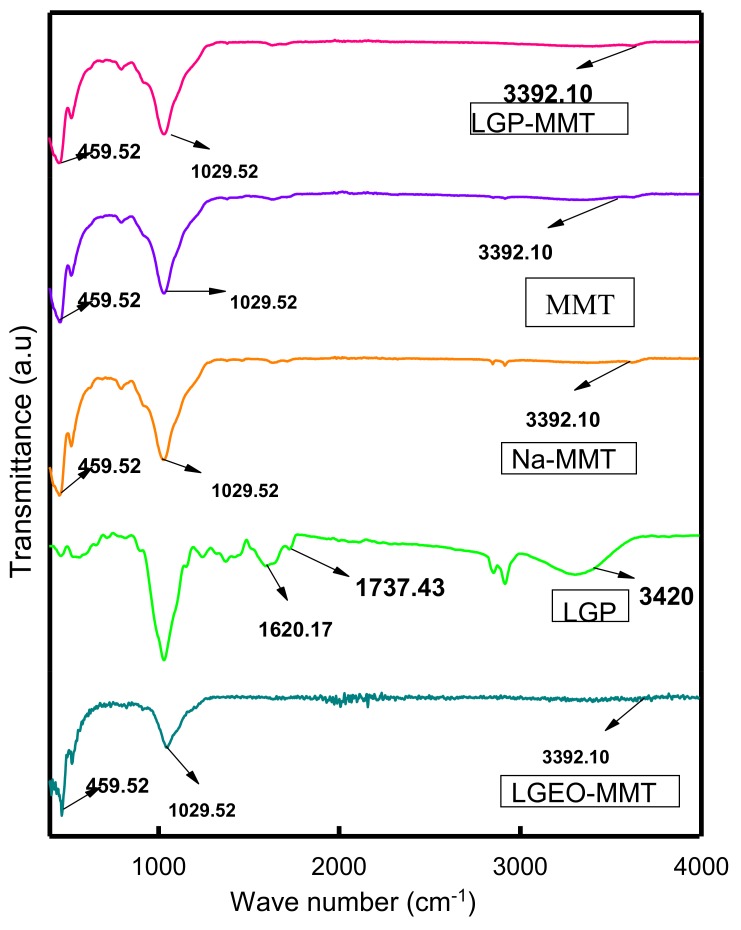
Fourier Transform Infrared (FTIR) spectra of LGEO-MMT, LGP, Na-MMT, MMT, and LGP-MMT.

The Infrared spectra of lemongrass essential oil-modified montmorillonite clay (LGEO-MMT), lemongrass powder (LGP), sodium montmorillonite (Na-MMT), montmorillonite clay (MMT), and lemongrass powder mixed with montmorillonite (LGP-MMT) are shown in Figure 3. There were visible bands at 459.52 and 1029.52 cm^−1^ and a little depression along 3392.10 cm^−1^ for all the treatments containing montmorillonite clay. The bands at 1737 cm^−1^, 1620 cm^−1^, and 1515 cm^−1^ in LGP, as shown in Figure 3, were attributed to the aliphatic, aromatic, and ester functional groups, respectively, which are present in the hemicellulose, wax, and pectin, as reported by Sun et al. [31]. The dominant spectrum at 3420 cm^−1^ corresponds to the O-H stretch, which is an aliphatic stretch in moieties of cellulose. The band at about 1739 cm^−1^ is due to the C=O stretching of the acetyl group and linked to the ester linkage of the carboxylic group of the ferulic acid and p-coumaric acid of lignin [31]. The broad band between 3200 and 3500 cm^−1^ represents that of an aliphatic compound attached to a hydroxyl (OH) group [32]. The OH functional group is usually present in alcohols, phenols, and carboxylic acids. However, the OH functional group in this spectrum neither belongs to a phenol nor a carboxylic acid because there is an absence of both benzene and carbonyl bands in the spectrum, respectively. The -OH in this spectrum belongs to alcohol. The IR spectrum of an alcohol is characterized by a strong and broad O-H stretch between 3300–3500 cm^−1^ [32]. The infrared (IR) spectrum of LGEO-MMT and Na-MMT, as shown in Figure 3, revealed that the peaks at 459 and 1029 cm^-1^ represent the bands for Si-O-Si bending and stretching, respectively [33]. These bands were present in two of the modified montmorillonite clays. The peaks in the region 3392 cm^−1^ represent the stretching of the -OH (hydroxyl group) of water within MMT [33]. Thus, Si-O bonds may be involved in the decontamination of T-2 by supplying energy to the van der Waals forces within the interlayer spaces of clay to enable the attraction of T-2 toxin.

### 2.3. The Decontamination of T-2 Toxin in Maize

**Figure 4:**
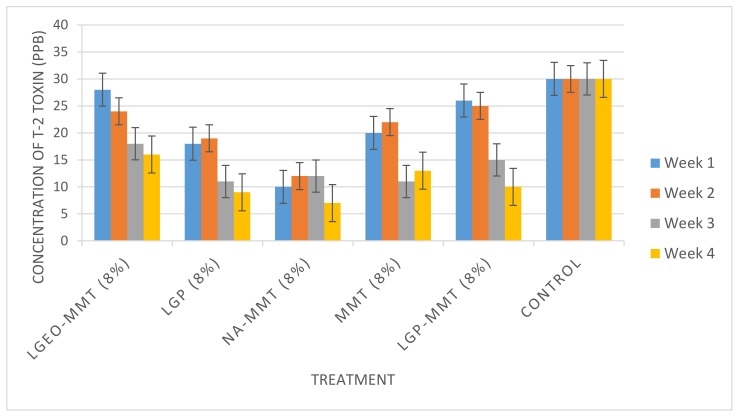
Evaluation of T-2 toxin after treatment with 8% of various types of modified clay for 4 weeks. LGEO-MMT: Lemongrass essential oil-modified montmorillonite clay; LGP: lemongrass powder; Na-MMT: montmorillonite clay washed with 1 mM NaCl; MMT: montmorillonite clay; LGP-MMT: lemongrass powder mixed with montmorillonite clay.

**Figure 5:**
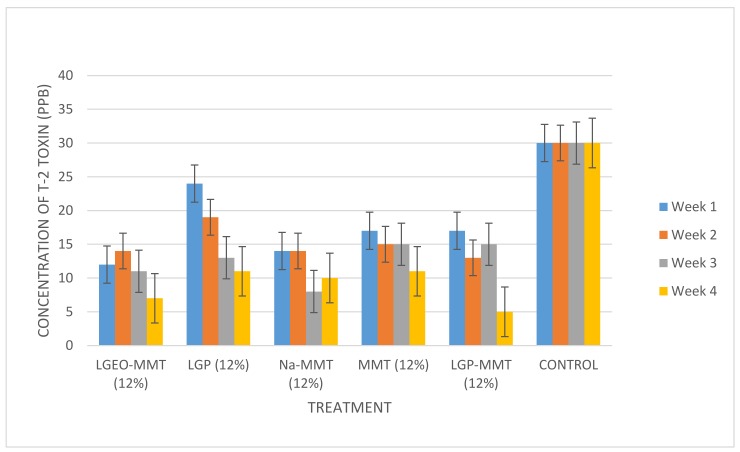
Evaluation of T-2 toxin after treatment with 12% of various types of modified clay for 4 weeks. LGEO-MMT: Lemongrass essential oil-modified montmorillonite clay; LGP: lemongrass powder; Na-MMT: montmorillonite clay washed with 1 mM NaCl; MMT: montmorillonite clay; LGP-MMT: lemongrass powder mixed with montmorillonite clay.

T-2 toxin had an apparent recovery of 116%. Its limit of detection (LOD) was 0.002 µg/kg, while its limit of quantification (LOQ) was 0.006 µg/kg. The various types of treatment (LGEO-MMT, LGP, Na-MMT, MMT, and LGP-MMT) applied to the maize sample from the same batch were effective over the treatment period of four weeks. The storage temperature (30 °C) was maintained for the treated maize samples and the control (untreated maize) throughout the four weeks of storage. The experiments were run for four weeks to establish the trend in the decontamination of T-2 toxin with time. The control (untreated maize stored under similar conditions as treated samples) remained unchanged throughout the treatment period from the first to the fourth week with a concentration of 30 ppb (Figure 4 and Figure 5).

The Post-Hoc analysis data generated showed significant differences among the five treatments against T-2 toxin. Further statistical analysis using LSD to compare mean values among various treatment formulations for the decontamination of T-2 toxin revealed that sodium montmorillonite (Na-MMT) was the most effective against T-2 toxin (*p* = 0.05). Montmorillonite clay, which has high adsorptive properties along with the positive charge of sodium (Na^+^), could have led to the effective trapping of T-2 toxin. It appears that the decontamination of T-2 toxin is better achieved by adsorption. Regular van der Waals gap between the sheets of clay is referred to as the interlayer spaces that possess a net negative charge due to ionic substitutions in the sheets of clay minerals [24]. Furthermore, the interlayer spaces can be assessed by water, organic cations, or polar organic liquids.

Sodium montmorillonite (Na-MMT) was more effective than the unmodified montmorillonite (MMT) due to the presence of Na^+^ ion, an alkali metal ion, which made the clay electrically neutral. Thus, the electrically neutral clay enhanced the binding of T-2 toxin. Silicates, which include montmorillonite clay being electrically negative, combine with ions, especially cations of alkali and alkali earth elements, to form electrically neutral species [34]. Lemongrass essential oil-modified montmorillonite clay (LGEO-MMT) at a concentration of 12% was the second most effective treatment against T-2 toxin. Hence, the decontamination of T-2 in maize was most effective throughout the period of four weeks in the following descending order: Na-MMT (8%), LGP-MMT (12%), MMT (12%), LGP (12%), and LGEO-MMT (12%), with 66%, 56%, 48%, 42%, and 37% decontamination, respectively.

For LGP-MMT at 12%, which was the second most efficient in the decontamination of T-2 toxin, the powder of *Cymbopogon citratus* in combination with montmorillonite clay contributed towards making montmorillonite clay more hydrophobic than the unmodified montmorillonite. Erminawati et al. [35] revealed that lemongrass contains hydrophobic compounds, including z-citral, estragole, borneol, methyl eugenol, beta-myrcene (MYR, 7-methyl-3-methylene-1,6 octadiene), geranyl acetate (3,7-dimethyl-2,6-octadiene-1-ol acetate), geraniol, limonene piperitone, citrat-2, citronellal, alpha-terpineole, proximal, pinene, farnesol, and (+)- cymbodiacetal [36]. Type A-trichothecenes (T-2 and HT-2) are non-polar, while Type B-trichothecenes (DON, NIV) are polar [37]. T-2, being a non-polar mycotoxin, attracts hydrophobic compounds. Hence, its ability to bind to the surface of the LGP-MMT, which contains hydrophobic compounds.

Microbiological approaches for the decontamination of T-2 toxin include the use of bacteria such as *Rhodococcus erythropolis*, *Rhodococcus globerulus, Rhodococcus rhodochrous,* and *Rhodococcus coprophilus*. These bacteria were 90% efficient in the degradation of T-2 toxin, although their degradation products were not studied [38]. Other bacteria such as species of *Pseudomonas*, *Arthrobacter*, *Blastobacter*, and those from the family Rhizobiaceae, have also been employed in the elimination of T-2 toxin with 100% efficiency recorded. However, Wachowska et al. [39] reported that degradation products such as Neosolaniol (NEO), T-2 tetraol, and T-2 Triol were produced. These T-2 degradation products and residual parent toxins are capable of inducing apoptosis [40]. Hence, the adsorption capacity observed in this study may be more advantageous because there are minimal chances for the formation of degradation products from T-2 toxin. Rather, the mycotoxin is adsorbed onto the surface of the modified montmorillonite clay, and the products formed are then excreted via feces. In addition, it is worth noting herein that the best way to protect foods and feeds against mycotoxins is to monitor their presence in foods and feeds. Therefore, regular routine analysis of food and feed for mycotoxins and control are recommended [41].

## 3. Conclusions

Montmorillonite clay was successfully modified on the surface with a sodium (Na^+^) ion, which made the clay electrically neutral and able to trap T-2 toxin especially, with sodium montmorillonite (Na-MMT) noted as the most efficient in decontaminating T-2 toxin in maize. Hence, the trapping of T-2 toxin was achieved by the regular van der Waals forces between the sheets of Na-MMT, which were enhanced by the Si-O bonds for the entrapment of this toxin within its layers. Adsorption processes for the decontamination of T-2 appear more promising than the biological methods for its decontamination because there could be very limited chances for the formation of toxic products.

## 4. Materials and Methods

### 4.1. Chemicals and Reagents

The chemicals used in this study were of analytical grade. These include: Methanol and Acetonitrile (LiChrosolv Reag. Ph Eur, Merck, Darmstadt, Germany, 99.9%), Formic acid (Fluka 56302-50ML-F, p. a, Thermofisher Inc, Oslo, Norway, 99.9%), Hexane (pro analyse, Merck, Darmstadt Germany, 99.9%); Acetone (HPLC grade, 99.8%, Sigma-Aldrich, Darmstadt Germany), Sodium chloride salt, and deionized water. T-2 mycotoxin standard was purchased from Sigma-Aldrich (Bornem, Belgium). Maize samples were purchased from one of the grain hubs in Ogun state, and a screening was performed to identify a maize sample that was naturally contaminated with T-2 toxin. The naturally contaminated T-2 maize sample was utilized for the experiment.

### 4.2. Sample Preparation

Montmorillonite K10 powder (CAS number 1318-93-0 (Sigma-Aldrich) was purchased, while *Cymbopogon citratus* (lemongrass) leaves used for the study were identified at the Botany Unit of the Department of Biological Sciences, Covenant University, Nigeria.

Montmorillonite clay K10 and lemongrass powder were the negative controls for the treatment. Fresh leaves of *Cymbopogon citratus* were gently washed with distilled water to remove dirt, and the leaves were allowed to air-dry at room temperature for 3 weeks. The dried leaves were ground into powder by using an electric blender (IKA M20, Wilmington, NC, USA). The lemongrass extracts were obtained by the Soxhlet extraction method described by Ojewumi et al. [42]. The dry powder of lemongrass (25 g) was extracted with 250 mL of hexane. The lemongrass extract was concentrated using a rotary evaporator (IKA RV10, Staufen, Germany) to obtain the essential oil used for the preparation of LGEO-MMT, which was done according to the method of Noudem et al. [43] with slight modification. All the formulations used for the decontamination of T-2 in the maize samples were prepared as described in Olopade et al. [44]. The montmorillonite clay was homogenized at 25 °C with a NaCl solution (1 mol L^−1^), at a solid:liquid ratio of 1/20. After that, the mixture was dialyzed in deionized water to eliminate chloride ions. The dialyzed samples were gently dried at 40 °C to allow for a gradual loss of water from the clay. In a 500 mL flask, 25 g of the clay powder was dispersed in 50 mL of essential oil solution (*v*/*v*) prepared with acetone. The mixture was whirled at room temperature (25 °C) in an overhead shaker at 50 revolutions per min. Afterward, the mixture was placed in a water bath operated at 60 °C for 90 min to allow for the evaporation of acetone. The mixture (LGEO-MMT) was dried overnight at 30 °C, and then stored in colored and tightly sealed vials using aluminum foil.

For the Na-MMT mixture, montmorillonite clay was modified by washing the clay with 1 mM NaCl solution in the ratio 1/20 (*w*/*v*), while LGP-MMT was prepared by mixing lemongrass powder with montmorillonite clay in the ratio 1:1.

### 4.3. Fourier Transform Infrared (FTIR) Spectroscopy

Fourier Transform Infrared (FTIR) Spectroscopy of the clay samples was performed with the aid of a Perkin Elmer Spectrum 100 FTIR Spectrometer. One drop of Nujol (a liquid hydrocarbon) was added to 1 mg of each sample and mixed thoroughly. The mull was then placed between sodium chloride plates, and the spectrum was recorded.

### 4.4. Scanning Electron Microscopy (SEM)

The samples were coated with carbon and examined with the aid of VEGA3 TESCAN scanning electron microscope at a voltage of 20 kV and magnification of 500× to examine their morphology.

### 4.5. Evaluation of T-2 Toxin before Treatment

For the method validation of T-2 toxin, the apparent recovery (AR) was obtained by spiking a known amount of T-2 toxin to a blank. The blank was spiked with 0.25 and 1 µL of the T-2 reference standard within a calibration range of 156.25–5000 µg/kg. The limit of detection (LOD) and limit of quantification (LOQ) were also determined.

Liquid chromatography-tandem mass spectrometry (LC-MS/MS) protocol, as reported by Sulyok et al. [45], was used to quantify T-2 toxin in maize before applying the various types of treatment (LGEO-MMT, LGP, Na-MMT, MMT, and LGP-MMT). In a 50 mL polypropylene tube (Sarstedt, Nümbrecht, Germany), 5 g of ground maize sample was extracted using 20 mL of an extraction solvent consisting of acetonitrile/water/formic acid (79:20:1, *v*/*v*/*v*) for 90 min on a GFL 3017 rotary shaker (GFL, Burgwedel, Germany). Five hundred µL of each filtered extract was then injected into the LC-MS/MS system to determine the level of T-2 toxin.

### 4.6. Decontamination of T-2 Toxin in Maize

Each of the various treatment formulations (LGEO-MMT, LGP, Na-MMT, MMT, and LGP-MMT) were applied to the maize sample in duplicates at 8% and 12% and stored for 4 weeks at a temperature of 30 °C in the incubator following the modified method of Atanda and Olopade [28]. A control was set up by subjecting the untreated maize sample from the same batch to the same condition of storage at 30 °C, just like the treated maize samples. LC-MS/MS protocol, as reported by Sulyok et al. [45], was used to quantify the levels of T-2 in the treated maize samples at the end of the first, second, third, and fourth weeks.

### 4.7. Statistical Analysis

The efficacy of the various types of treatment, which were applied in duplicates, was determined by Post-Hoc analysis using the IBM SPSS statistics software (ver. 23, 2015 Inc., Chicago, IL, USA). Subsequently, mean values among the various treatment formulations for the decontamination of T-2 toxin were compared by the Least Significant Difference (LSD) test and considered to differ at *p* ≤ 0.05.

## Abbreviations

Bw	body weight
LGEO-MMT	lemongrass essential oil-modified montmorillonite clay
LGP	lemongrass powder
Na-MMT	montmorillonite clay washed with 1 mM NaCl
MMT	montmorillonite clay
LGP-MMT	lemongrass powder mixed with montmorillonite clay
FTIR	Fourier Transform Infrared Spectroscopy
LSD	Least Significant Difference
AF	Aflatoxin
NEO	neosolaniol
DON	deoxynivalenol
FB_1_	fumonisin B_1_
NIV	nivalenol
ZEA	zearalenone
